# Does attractiveness influence condom use intentions in heterosexual men? An experimental study

**DOI:** 10.1136/bmjopen-2015-010883

**Published:** 2016-06-17

**Authors:** Anastasia Eleftheriou, Seth Bullock, Cynthia A Graham, Nicole Stone, Roger Ingham

**Affiliations:** 1 Department of Electronics and Computer Science, University of Southampton, Southampton, UK; 2 Institute for Complex Systems Simulation, University of Southampton, Southampton, UK; 3 Department of Computer Science, University of Bristol, Bristol, UK; 4 Centre for Sexual Health Research, Department of Psychology, University of Southampton, Southampton, UK

**Keywords:** STIs, sexual health, condoms

## Abstract

**Objectives:**

Judgements of attractiveness have been shown to influence the character of social interactions. The present study sought to better understand the relationship between perceived attractiveness, perceived sexual health status and condom use intentions in a heterosexual male population.

**Setting:**

The study employed an electronic questionnaire to collect all data, during face-to-face sessions.

**Participants:**

51 heterosexual, English-speaking men aged between 18 and 69 years.

**Outcome measures:**

Men were asked to rate the attractiveness of 20 women on the basis of facial photographs, to estimate the likelihood that each woman had a sexually transmitted infection (STI) and to indicate their willingness to have sex with or without a condom with each woman.

**Results:**

The more attractive a woman was judged to be on average, the more likely participants would be willing to have sex with her (p<0.0001) and the less likely they were to intend to use a condom during sex (p<0.0001). Multivariate analysis revealed that higher condom use intentions towards a particular woman were associated with lower ratings of her attractiveness (p<0.0005), higher ratings of her STI likelihood (p<0.0001), the participant being in an exclusive relationship (p=0.002), having a less satisfactory sex life (p=0.015), lower age (p=0.001), higher number of sexual partners (p=0.001), higher age at first intercourse (p=0.002), higher rates of condomless sex in the last 12 months (p<0.043) and lower confidence in their ability to assess whether or not a woman had an STI (p=0.001). The more attractive a participant judged himself to be, the more he believed that other men like him would engage in condomless sex (p=0.001) and the less likely he was to intend to use a condom himself (p=0.02).

**Conclusions:**

Male perceptions of attractiveness influence their condom use intentions; such risk biases could profitably be discussed during sex education sessions and in condom use promotion interventions.

**Strengths and limitations of this study**

- First study to explore the relationship between perceived attractiveness and condom use intentions in heterosexual men.
- Findings extend the literature by investigating the association between own perceived attractiveness, sex and condom use intentions.
- Small and relatively homogenous sample.
- Reported condom use intentions may or may not reflect actual condom use behaviour.
- Findings inform interventions for public health.

## Introduction

The impression that a person's appearance makes strongly influences their interactions within their social environment. Facial attractiveness, in particular, has been the subject of extensive research in the human behavioural sciences as it dramatically influences social experience,^1^ including decisions about sexual behaviour.^2^^–^4

Recent evidence by Nedelec and Beaver^5^ supported the hypothesis that there is an association between perceived physical attractiveness and health. Specifically, their findings, which were consistent across men and women, showed that the more attractive a person was rated by participants, the less likely they were to be diagnosed with a neuropsychological disorder or a chronic disease. Although some chronic diseases can affect an individual's attractiveness directly (eg, by affecting the skin), other conditions, such as the majority of sexually transmitted infections (STIs), might not necessarily be expected to impact on a person's attractiveness directly. Despite this, it has been shown that people feel that they are able to judge the presence or absence of an STI/HIV on the basis of visual appearance alone.^6^

Lennon and Kenny^7^ reported that women's physical attractiveness ratings of men are a strong positive predictor of the women's willingness to have unprotected sex, even when women believed that attractive men were more likely to have an STI. Women in this study indicated that they preferred their partners to be attractive even though this might put their sexual health at risk. As an alternative to simple ratings of attractiveness and willingness to have sex, Rupp *et al*^8^ used fMRI to measure the brain activity of 12 single heterosexual women, none of whom were using any hormonal contraception, while they viewed photographs of male faces. These stimuli were paired with information regarding the potential health risk posed by each man as a sexual partner, in the form of his number of sexual partners and his frequency of condom use. Participants showed a sexual preference for low-risk men rather than high-risk men. However, in a similar study from the same research group,^9^ the same 12 female participants judged men with masculinised faces to be riskier and more attractive than those with feminised faces.

Fishbein *et al*^10^ and Henderson *et al*^11^ focused on the association between romantic attraction and health risks by asking male and female participants to rate attributes that are often used to describe romantic partners, such as ‘physical build’ or ‘emotionality’, on their importance for partner selection. These studies reported that the more a participant was attracted to a person with ‘risky’ features, the less likely they were to consider that the person presented a health risk. Also, high sensation seekers rated potential partners as more attractive and less risky than low sensation seekers did. However, these studies did not address the effect of the participants' own perceived attractiveness on their judgements of risk and attraction and did not consider how these judgements related to condom use intentions in the context of casual sex.

Although there is consistent evidence of links between attractiveness and sexual behaviour, the mechanisms underlying these relationships have not been elucidated. Another unexplored issue is whether the relationships between attractiveness and sexual behaviour differ by gender.

The current study focused on how the perceived facial attractiveness of women by heterosexual men affected their willingness to have condomless sex and perceptions of STI risk. A similar work by Dijkstra *et al*^12^ found that 72 male undergraduates asked to rate pictures of women and consider a brief description of their personality were more motivated to have sex with a physically attractive woman, even though they believed that she was more likely to have an STI. However, condom use intentions were not evaluated. Agocha and Cooper^13^ did address this issue directly, finding that physical attractiveness was not a direct predictor of condom use intentions in a sample of psychology undergraduates. However, path analysis revealed that the total indirect effects of physical attractiveness on intentions to use condoms were five to six times larger than those for sexual health information about the target. More recent work by Epstein *et al*,^14^ which involved an internet study displaying a picture and a biography for a randomly assigned target, also supported the hypothesis that a potential partner's physical attractiveness has an impact on intentions to have sex in men and women. However, no significant direct effects of physical attractiveness on intentions to have condomless sex or on perceived STI risk were found.

Although the above studies give some insights into the relationships between facial attractiveness, perceived risk and condom use intentions, their findings were not entirely consistent; in many cases, only one or two pictures of the opposite sex were rated by participants, and not all of the studies considered the context of demographic variables and sexual history. The current study extends research in this area by eliciting men's condom use intentions towards 20 women, and by evaluating these not only with respect to the perceived attractiveness of the women, but also the participants' perceptions of their own attractiveness, their sexual history, including their typical condom use behaviour, and their perceptions of other men's condom use intentions.

It is important to consider participant's self-rated attractiveness when analysing condom use intentions, since self-perceived attractiveness may influence sexual preferences,^15^ perceived STI risk^12^ and also mating decisions, as individuals tend to choose partners who physically resemble them or appear to have similar facial features.^16^ Moreover, eliciting participants' judgements regarding the condom use intentions of other men like themselves may address possible demand characteristics of the study situation, which can encourage participants to provide a ‘correct’ response to questions directly targeting their own sexual behaviour.^17^ ^18^ Finally, in order to consider the possible influence of demographics and sexual experience on condom use intentions, the possible effects of participant age, satisfaction with their sex lives, their number of sexual partners and the age of their first sexual intercourse should be explored. All of the aforementioned variables were addressed in the current study.

The primary purpose of the current study was better to understand the relationship between perceived attractiveness and condom use intentions in heterosexual men and to gain insights into the relationship between perceived attractiveness, demographics, sexual history and perceived sexual health status. The research questions were: (1) does the perceived attractiveness of a potential sexual partner affect sex and/or condom use intentions? (2) does a participant's own perceived attractiveness affect their sex and/or condom use intentions? (3) does heterogeneity in the association between perceived sexual health status and perceived attractiveness influence condom use intentions? (4) Do demographic or sexual experience variables predict condom use intentions?

## Methods

### Participants

Data were collected at the University of Southampton between January and May 2015. Men in Southampton and surrounding areas were recruited via social media (Facebook and Twitter), posters at the University and on community advertisement boards, and advertisements on the University's online participant recruitment site (eFolio). Potential participants were informed that data would be collected using questionnaires in order to investigate the influence of attractiveness on sexual attitudes and intentions and they were screened for eligibility via email. Inclusion criteria were: 18–69 years of age; English-speaking and heterosexual man. Fifty-one men were screened and all met the inclusion criteria. All of them agreed to participate in a face-to-face session in a university location and provided electronic informed consent.

### Measures

The study employed an electronic questionnaire to collect all participants' data. A draft questionnaire was initially trialled on five pilot study participants and was then refined on the basis of their feedback during individual think aloud sessions, in which they explained what they could and could not understand and also how participation made them feel. The final questionnaire comprised three sections: (1) participants' demographic information and judgement of their own attractiveness, (2) information regarding the participant's own sex life, (3) 5 judgements on each of 20 women on the basis of a single full frontal facial photograph. The order of the 100 test items in the third section was fully randomised for each participant.

In the rest of the paper, we use a series of single-letter labels to identify key variables associated with six categories of questionnaire items introduced in parentheses on their first mention below.

#### Demographics and own attractiveness

Participants were asked about their age, ethnicity and occupation, and then asked to rate their own attractiveness (O) on a scale from 0 to 100, with 0 indicating ‘very unattractive’ and 100 indicating ‘very attractive’.

#### Sexuality variables

Participants' satisfaction with their sex life was assessed by the following item, “Thinking about your sex life in the last year, how much do you agree or disagree with the following statement: ‘I feel satisfied with my sex life’”. Response options ranged from ‘1’ (strongly agree) to ‘5’ (strongly disagree).

Participants also indicated whether they were attracted to men, women, either or both, their relationship status and how many lifetime sexual intercourse partners they had had. Three further yes/no questions were asked: “As far as you know, have you ever had an STI?”, “As far as you know, do you currently have an STI?” and “As far as you know, are you allergic or sensitive to latex, non-latex condom and/or lubricants?” Finally, participants were asked: “Which one of the following percentages describes better the proportion of occasions of intercourse you have not used a condom in your lifetime?”, “Which one of the following percentages describes better the proportion of occasions of intercourse you have not used a condom in the past twelve months?” and “How easy would it be for you to identify whether a woman has an STI, without asking?” Answers ranged from 0% to 100%, in six intervals with boundaries at 10%, 30%, 50%, 70%, 90% and 100%.

#### Ratings of facial photographs

Participants were asked to provide 5 ratings for each of 20 women on the basis of a single black and white photograph of the woman's face taken from the Extended Cohn-Kanade (CK+) database:^19^ “Please rate the attractiveness of the following woman” (A); “If you were single, how likely would you be to have sex with this woman should the opportunity arise?” (S); “If you were single and you were to have sex with this woman, how likely is it that you would use a condom?” (C); “Out of 100 men like you, how many would have condomless sex with this woman should the opportunity arise?” (M) and “How likely is this woman to have an STI?” (I). Participants indicated their answer to each question by moving a slider between 0 and 100. These 100 items were presented in fully randomised order, that is, the 5 questions regarding a particular woman were not presented together or in a particular order and, similarly, the 20 questions regarding a particular rating (eg, attractiveness) were not presented together or in a particular order. Prior to commencing the task, a simultaneous presentation of all 20 faces was shown to enable the participants to anchor their judgements.

#### Stimuli selection and procedures

The Extended Cohn-Kanade (CK+) database^19^ includes facial image data from 210 men and women aged 18–50 years. The data set includes 81% Euro-American, 13% Afro-American and 6% ‘other’ participants. For this study, 20 female faces with neutral expression were chosen at random and were displayed in black and white.

### Procedure

After providing electronic informed consent, each participant completed the self-administered electronic questionnaire on a university computer (taking between 25 and 30 min). A researcher was present during the session in case the participant needed clarification of any questions, but they were not able to see participants' responses. Each participant received £4 (∼6US$) at the end of the session. The Ethics Committee of the University of Southampton approved the protocol (ERGO ref: 13607).

### Data analysis

To identify factors influencing condom use and interactions among them, a series of bivariate associations (Pearson's correlation coefficients^i^) were calculated, followed by a multivariate test of associations (a repeated-measures linear mixed model).

## Results

### Demographics

Fifty-one heterosexual men, mean age 26.41 years (SD=7.91, minimum=19, maximum=61), completed the session. Twenty participants were white British, 17 were white ‘other’ (eg, Italian) and the remaining were identified as Indian, Chinese, any other Asian background, Caribbean, Hispanic and other mixed background.

### Sexual experience variables

In response to the statement: “I feel satisfied with my sex life”, 5 (9.8%) participants agreed strongly, 25 (49%) agreed, 10 (19.6%) neither agreed nor disagreed, 8 (15.6%) disagreed, 2 (4%) disagreed strongly and 1 (2%) preferred not to say. All of the participants reported that they were exclusively attracted to women except one who reported that he was attracted to men and women. Twenty-three (45.1%) participants were single, 21 (41.2%) were in an exclusive relationship, 4 (7.8%) were in an open relationship and 3 (5.9%) were married. None reported having an STI at the time of the session, and 5 (9.8%) participants reported having an STI in the past. The average number of lifetime sexual partners was 9.65 (SD=10.95, minimum=0, maximum=60) and the mean age at first sexual intercourse was 18.3 years (SD=3.4, minimum=14, maximum=30). None of the participants reported suffering from an allergy to latex, non-latex condoms and/or lubricants. Reported rates of condomless sexual intercourse are presented in table 1.

**Table 1:** The percentage of sexual intercourse episodes in which condoms were not used reported by 47 participants (ie, excluding four participants who had never had sexual intercourse) during their lifetime and during the last 12 months

% Condomless sex	<10%	<30%	<50%	<70%	<90%	≤100%
Lifetime	14	6	7	11	5	4
Past 12 months	20	2	6	4	4	11

### Participants' ratings

In analysing participants' ratings, we distinguished between considering the data associated with each of the women being rated, aggregating over the participants' ratings and considering the data associated with each participant, aggregating over the women that he rated. For example, were some women judged to be more attractive than others on average, considering the participants as a group? This set of values will be denoted as 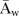 (see table 2 for ratings). Alternatively, did some participants find the set of 20 women in the study more attractive than other participants did, considering the women as a group? This set of values will be denoted as 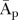.

**Table 2:** The mean participant ratings for each female photograph (scale 0–100)

Woman	Attractiveness ( 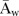 )	Condom use intentions ( 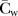 )	Sexually transmitted infection likelihood ( 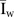 )	Other men: sex without a condom ( 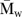 )	Willingness to have sex ( 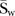 )
1	30.5	87.3	44.9	27.2	31.9
2	18.7	89.0	32.2	19.8	16.5
3	38.3	86.7	36.9	27.8	33.1
4	40.7	83.4	26.1	29.9	42.3
5	40.9	85.2	33.3	31.3	41.2
6	46.3	85.9	28.5	34.4	45.6
7	69.5	82.1	35.7	46.4	71.3
8	69.1	78.0	24.8	46.0	66.2
9	45.7	84.9	31.8	34.6	46.0
10	53.8	77.3	27.2	34.6	54.4
11	55.5	81.3	24.8	39.1	58.5
12	52.7	82.9	37.4	34.3	56.0
13	47.4	87.2	32.7	31.8	45.4
14	45.2	83.7	27.2	33.1	46.4
15	40.9	86.2	29.7	30.9	39.9
16	35.7	89.5	37.0	29.1	32.1
17	30.5	85.3	30.6	26.3	29.3
18	47.6	83.9	33.8	30.7	49.5
19	46.5	83.0	33.8	33.5	42.7
20	56.2	81.7	28.1	37.4	58.2

#### Associations between participants' ratings of the 20 women

First, we constructed average ratings for each woman and considered relationships among these. The more attractive a woman was judged to be on average, 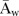, the more likely participants would be willing to have sex with her, 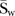 (r=0.985, df=18, p<0.0001). Furthermore, the more attractive a woman was judged to be, 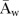, the less likely men were to intend to use a condom during sex, 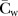 (r=−0.785, df=18, p<0.0001). Consequently, average condom use intentions, 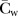, tended to be lower for women that participants were, on average, more willing to have sex with, 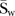 (r=−0.795, df=18, p<0.0001).

On average, participants judged that more men like themselves would have sex without a condom, 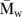, to a greater extent with women that the participants judged, on average, to be more attractive, 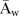 (r=0.970, df=18, p<0.0001), and with whom the participants were, on average, more willing to have sex, 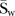 (r=0.952, df=18, p<0.0001). Consequently, where the average judgement of the number of men willing to have condomless sex with a woman, 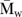, was high, participants' average condom use intentions towards the woman, 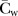, were lower (r=−0.730, df=18, p<0.0001).

Although the average perceived STI likelihood for a woman, 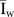, was positively correlated with average condom use intentions towards her, 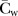 (r=0.553, df=18, p<0.05), it had no significant association with her average perceived attractiveness, 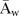, or with participants' average willingness to have sex with her, 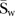. These bivariate associations are summarised in table 3.

**Table 3:** Bivariate associations between mean ratings for 20 women (df=18) of their attractiveness, 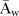, condom use intentions towards them, 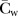, their sexually transmitted infection likelihood, 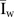, the extent to which men like the participants would be willing to engage in condomless sex with them, 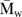 and the willingness of the participants to have sex with them, 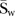

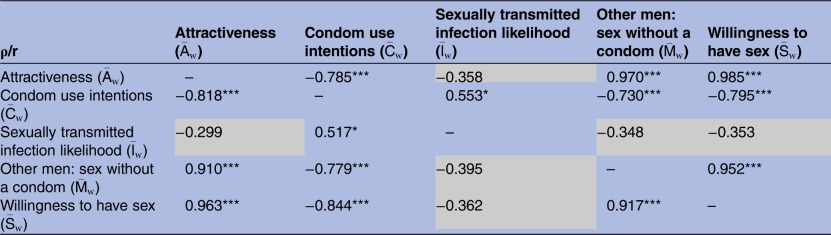

Pearson's r values are shown in the upper right half of the table, Spearman's ρ in the lower left: *p<0.05, **p<0.01, ***p<0.001, grey cells=NS.

#### Overall ratings of women

Next, for each participant, we averaged over their ratings of the 20 women and considered relationships among these ‘overall’ ratings. Participants who tended, overall, to rate the 20 women as more attractive, 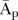, tended to be more willing to have sex, overall, 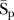 (r=0.855, df=49, p<0.0001). Participants who judged that men like themselves were more willing, overall, to have condomless sex with the 20 women, 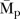, also tended to believe that, overall, the 20 women had a higher likelihood of having an STI, 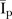 (r=0.544, df=49, p<0.001) and themselves had higher overall condom use intentions, 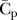 (r=0.313, df=49, p<0.05). However, overall judgement of STI likelihood was not related to overall condom use intentions. These relationships are summarised in table 4.

**Table 4:** Bivariate associations (Pearson's r) between 51 (df=49) participants' overall ratings

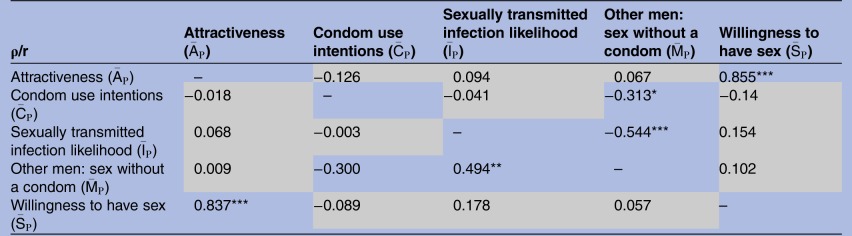

Significance levels are indicated: *p<0.05, **p<0.01, ***p<0.001, grey cells=NS.

#### Influence of perceived own attractiveness and ability to detect STIs

The more attractive a participant judged himself to be, 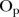, the more he believed that, overall, other men like him would not use a condom during sex, 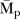 (r=0.491, df=40, p=0.001) and the less likely he was, overall, to intend to use a condom himself, 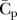 (r=−0.355, df=40, p=0.02).

Participants' confidence in their ability to detect whether a potential sexual partner had an STI without asking was significantly negatively correlated with their overall tendency to rate women as more attractive, 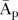 (r=−0.295, df=49, p=0.036), and more likely to have an STI, 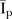 (r=0.323, df=49, p=0.02), and was also associated with overall lower condom use intentions in themselves, 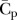 (r=−0.403, df=49, p=0.003), and men like themselves, 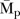 (r=0.292, df=49, p=0.038). Participants who were more confident in their ability to detect STIs without asking also tended to rate themselves as more attractive (r=0.612, df=40, p<0.0001).

#### Influence of age and sexual experience variables

Participants more satisfied with their sex life tended to provide lower overall attractiveness ratings, 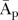 (r=0.373, df=49, p=0.006). Neither a participant's age, number of lifetime sexual partners, nor their relationship status, had an association with their overall ratings. Of the 47 participants who had indicated that they had experienced sexual intercourse, those who reported having had an STI gave higher overall ratings of attractiveness, 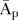 (r=0.346, df=45, p=0.017), willingness to have sex, 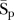 (r=0.308, df=45, p=0.035) and rates of condomless sex in men like themselves, 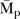 (r=0.312, df=45, p=0.016). Age at first sexual intercourse and rate of condomless sex over the last 12 months were not significantly related to the participants' overall ratings, but participants' lifetime rate of condomless sex was negatively associated with overall condom use intentions towards the women that they rated in the study, 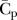 (r=−0.301, df=45, p=0.04). These relationships are summarised in table 5.

**Table 5:** Bivariate associations (Pearson's r) between 51 (df=49) participant demographic and sex experience variables (left column) and their mean ratings of 20 women

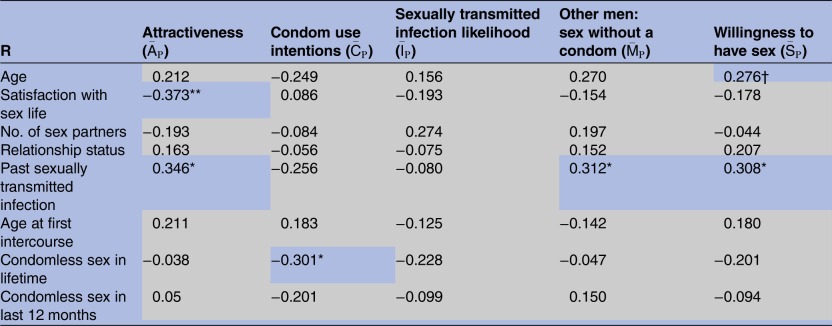

Four participants indicating that they had not had sexual intercourse were excluded from the bottom four analyses (ie, df=45).

Significance levels are indicated: *p<0.05, **p<0.01, grey cells=NS.

†Although r is significant (at p<0.05) for Age by 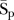, Spearman's ρ (0.04) is not significant (p=0.78), suggesting that outlier participants have had a disproportionate influence on the association.

#### Linear mixed model

A linear mixed model with repeated measures was constructed in order to carry out a multivariate analysis addressing the question: what linear combination of factors best explains the variation in participants' condom use intentions across the 20 women rated. The main benefit of a linear mixed model is that it enables repeated measures to be handled (in this case the 20 women rated), and deals with the possibility that participants may vary in the overall level and variability of their condom use intentions.

The set of participant condom use intention ratings (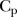) was the outcome variable, with the repeated measures being the individual women rated. Four participants who had indicated that they had not had sexual intercourse were excluded in order to include sexual experience variables related to sexual activity (eg, age at first sexual intercourse). All demographic and sexual experience variables and rating variables were included as main effects, except those for which there was no variation in the participant sample (ie, allergy to latex and current STI)^ii^. Willingness to have sex ratings was excluded from the model due to their very strong collinearity with attractiveness ratings (r=0.8). The model thus attempted to identify a single set of relationships that could account for all participants' patterns of condom use intentions.

Variables significantly associated with higher condom use intentions towards a woman were lower ratings of her attractiveness, 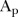 (p<0.0005), higher ratings of her STI likelihood, 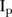 (p<0.0005) and lower estimates of the number of men who would have condomless sex with her, 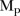 (p<0.0005). Demographic and sex experience variables that were significantly associated with participant's reporting higher condom use intentions were being either married or in an exclusive relationship (p=0.002), being less satisfied with sex life (p=0.016), lower age (p=0.001), higher number of sexual partners (p=0.001), higher age of first intercourse (p=0.003), lower lifetime rates of condomless sex (p<0.0005) but higher rates of condomless sex in the last 12 months (p<0.041) and lower confidence in their ability to determine, without asking, whether a woman had an STI (p=0.001). The participant's STI history was not significant.

#### Participant heterogeneity analysis

Note that while the above analyses have revealed relationships between average ratings, they are quite insensitive to between-participant heterogeneity. This means that they are not suited to answering questions such as: do the condom use intentions of participants who are more attracted to women that they regard as at higher risk of an STI differ from those of participants who are attracted to ‘safe’ women? The following analyses address this deficiency by considering within-participant correlations between ratings (eg, the correlation between condom use intentions and STI risk for each participant).

Some within-participant correlations were very consistent, for example, the average correlation between participant's attractiveness ratings and their willingness to have sex ratings was 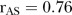, with 45 participants exhibiting a correlation >0.8. However, in other respects, participants were more heterogeneous. In particular, while the average correlation between participant's attractiveness ratings and their STI likelihood ratings was close to zero, 21 participants had strong preferences for either ‘safe’ or ‘risky’ women, with 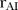 correlations either greater than 0.4 or less than −0.4.

**Figure 1:**
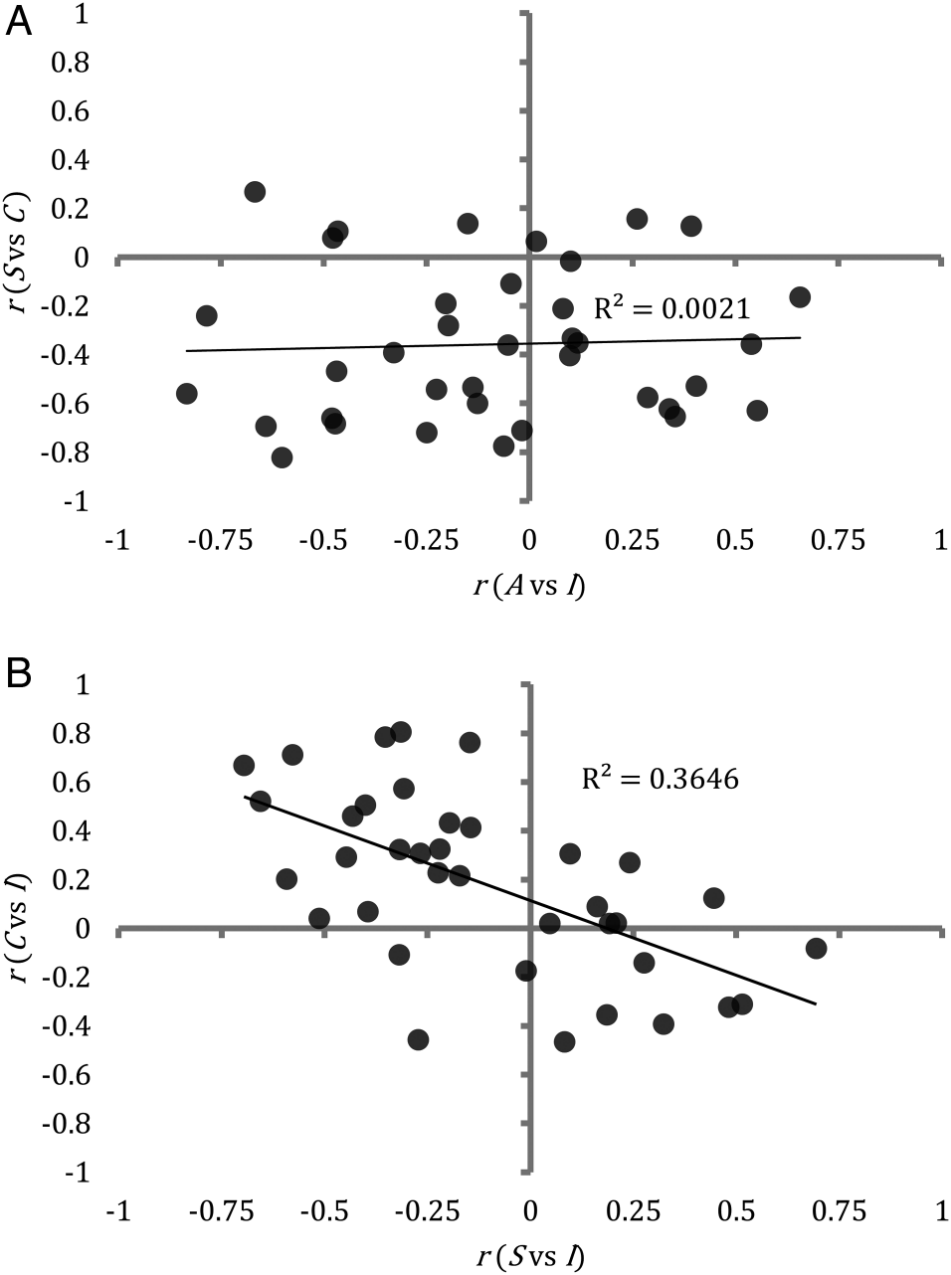
Scatterplots showing trends in the ratings of individual participants. Each point represents data from one participant: (A) the extent to which a participant tended to be attracted to women whom he judged to be likely to have a sexually transmitted infection (horizontal axis) had no influence on the extent to which he intended to use condoms with women he was willing to have sex with. (B) The extent to which a participant tended to be more willing to have sex with women whom he judged to be more likely to have asexually transmitted infection (horizontal axis) was significantly associated with his tendency to have lower condom use intentions towards those high-risk women (R^2^=0.3646, df=49, p<0.0001).

The extent to which a participant was attracted to more risky sexual partners (ie, the correlation between participant's attractiveness ratings and their STI likelihood ratings) had no influence on the correlation between their condom use intentions ratings and their willingness to have sex ratings. Men who were attracted to high-risk women and men who were attracted to low-risk women both had lower condom use intentions towards the women that they were attracted to (cf. the flat distribution of points in the lower half of figure 1A). Consequently, participants more willing to have sex with safer women had lower condom use intentions towards safer women, whereas participants more willing to have sex with riskier women tended to have lower condom use intentions towards those high-risk women (cf. the diagonal distribution of points in figure 1B, with risk seekers in the bottom right quadrant).

## Discussion

This study suggests that there is a strong correlation between perceived attractiveness and condom use intentions in heterosexual men. Participants were more willing to have sex with attractive women, but were less inclined to use condoms when they do so. Agocha and Cooper^13^ found that male participants perceived women of high attractiveness as less risky and reported that they were less likely to discuss risk-relevant topics with them. Conceivably, such men might believe that attractive women take care of themselves more than less attractive women do, and therefore that they are healthier and pose less of a health risk, legitimising their reduced condom use intentions. However, this hypothesis is undermined by Dijkstra et al's finding^12^ that participants perceived highly attractive women to be more promiscuous and more likely to have an STI than less attractive women. Conversely, Epstein *et al*^14^ did not find a significant effect of perceived attractiveness on condomless sex intentions or perceived STI likelihood.

It seems possible that these diverse findings stem from genuine diversity in the male population. The current study found no overall relationship between judgements of STI likelihood and judgements of attractiveness. On average, men are not more attracted to women they judge to be at lower risk of STI. Instead, participants varied significantly in this respect, with some men significantly more attracted to women that they judged to be free of STIs and some men significantly more attracted to women that they judged to be more likely to have an STI. If condom use intentions in men reflect their judgements of STI risk, then we might expect these intentions to differ along this risk-seeking/safe-seeking dimension. This is, however, not the case. Men who are more attracted to ‘riskier’ women are just as disinclined to wear a condom when they have sex with these women as men who are more attracted to ‘safer’ women. This leads to behaviour that appears irrational from the perspective of avoiding infection: men attracted to riskier women are more inclined to use condoms with the safer women who they are less attracted to, rather than the risky women with whom they are more willing to have sex with. This underlines the fact that people are often fully aware of the ‘rational’ responses (in a health promotion sense), but their actual behaviour does not necessarily follow suit.^20^

The tendency of participants to have reduced condom use intentions towards women with whom they are willing to have sex is surprising in the light of their judgement that a greater number of men like themselves would be willing to have condomless sex with these women, which implies that these women are at high risk of STIs. This observation did not translate into higher perceived risk, either in terms of increased overall condom use intentions towards more ‘attractive’ women or increased overall expectations of infection in ‘attractive’ women. This finding agrees with Fishbein *et al*,^10^ who found that risk information about a partner is sometimes ignored when the partner is attractive.

This study also sheds some light on the sexual risk taking of men based on their own perceived attractiveness. Men who judged themselves to be more attractive were less likely to intend to use a condom and also estimated higher rates of condomless sex in men like themselves. This is unlikely to be due to these men having had more sexual experiences than men who are less confident of their attractiveness, since reporting a high number of sexual partners was associated with higher condom use intentions. Alternatively, attractive men may feel that they can influence their partner not to use a condom to a greater extent than less attractive men, who might be more worried that if their partner does not agree to condom use, they might not have a high chance of success with them or other women.

Studies have demonstrated that people form beliefs about STI risk during first encounters,^21^ that these judgements can be made within milliseconds^22^ and that they are based on a wide variety of factors.^23^ However, prior to this study, the influence on condom use intentions of participants' confidence in their judgements had not been thoroughly investigated. Participants' confidence in their ability to judge whether a potential sexual partner had an STI on the basis of appearance was found to be significantly positively correlated with participants' tendency to rate women as less attractive and as more likely to have an STI, and with lower condom use intentions in themselves and men like themselves, and higher self-perceived attractiveness.

Condom use intentions were positively correlated with reported lifetime condom use, which suggests that participants responded to the hypothetical survey scenarios in a manner that reflected to some extent their real sexual behaviour.

Although we might have expected to find little variability in participants' ratings in a study with strong normative demand characteristics (eg, participants might feel that they are expected to use condoms when they have sex with women), the data revealed a wide variety of behaviour and intentions, organised around strong trends and patterns despite the relatively small sample. Moreover, men varied considerably in their attitudes to sexual behaviour, condom use and risk. This suggests that tailored sex education interventions, to target particular groups of people, might be useful; for example, a message that is appropriate for men who report that they are attracted to women who are likely to be free from infection may not be effective for men who are attracted to women that they believe are more likely to have an STI (figure 1). More generally, it may be profitable to explore interventions that target the tensions between some of the beliefs exhibited by the participants here; for instance, the fact that participants believed that many men like themselves would most like to have unprotected sex with the kind of women that the participants themselves find attractive. This intervention could take the form of a computer game, which adapts its content based on the target group or individual. As younger people are very familiar with computer and video game playing, they may find it easier to engage with this kind of sex education intervention and, therefore, they could better understand the risks and their misconceptions.^24^ Education through games can be effective as it is predominantly the player who directs activity and therefore the learners are involved in the learning process, in contrast with traditional education, which suggests a teacher-centred approach where learners are relatively passive.

Future research could also investigate whether individual differences in variables known to influence risk taking, such as sexual sensation seeking^25^ and sexual excitation/inhibition,^26^ might mediate the relationship between attractiveness and condom use intentions.

### Limitations

Participants completed the study in the presence of a female researcher who may have influenced their responses, as has been shown in previous studies.^27^ Future studies should control for this possible confounding effect. Also, the degree to which participants were sexually aroused was not recorded during the study. Sexual arousal could dramatically influence their condom use intentions.^28^ Another limitation was the small and relatively homogeneous sample; however, 51 men and 20 stimulus women provided over 1000 data points for each measure. Finally, participants' reported condom use intentions in this study may or may not resemble their actual usual condom use behaviour since condom use behaviour may not correlate highly with condom use intentions^29^ due to the influence of contextual factors such as alcohol and sexual arousal.

Notwithstanding these limitations, this study is the first to explore the relationship between perceived attractiveness and condom use intentions in heterosexual men, including their self-ratings of attractiveness and previous sexual experiences.

## Conclusions

In summary, this study extends the literature by investigating the association between own perceived attractiveness, sex and condom use intentions. Additionally, the associations between age, sex life satisfaction, STI history, reported condom use with sex and condom use intentions were explored. Male perceptions of attractiveness influence their condom use intentions; such risk biases could profitably be discussed during sex and relationships education sessions in educational settings.

## Footnotes

i Spearman's ρ was also calculated and provided similar values unless explicitly noted.

ii Twelve variables were included in the mixed linear model: participants' ratings of women's attractiveness, STI likelihood and the extent to which other men like themselves would be willing to have condomless sex with the women, as well as participant age, satisfaction with their sex life, relationship status, STI history, number of sexual partners, age at first intercourse, lifetime condom use, condom use in the past year and their confidence in their ability to predict a woman's STI status on the basis of her appearance. The “participant's own attractiveness” variable was not included due to missing data for the first nine participants who did not receive this questionnaire item.
